# Developing a male-specific counselling curriculum for HIV treatment in Malawi

**DOI:** 10.12688/gatesopenres.16357.1

**Published:** 2025-07-28

**Authors:** Misheck Mphande, Isabella Robson, Julie Hubbard, Eric Lungu, Elijah Chikuse, Khumbo Phiri, Morna Cornell, Sam Phiri, Thomas J Coates, Kathryn Dovel

**Affiliations:** 1 Partners in Hope, Lilongwe, Malawi; 2 University of California Los Angeles David Geffen School of Medicine, Los Angeles, California, USA; 3 University of Cape Town School of Public Health, Observatory, Western Cape, South Africa; 4 University of California Global Health Institute, San Francisco, California, USA

**Keywords:** Men, HIV, antiretroviral therapy, counselling, curriculum, male-specific, sub-Saharan Africa

## Abstract

**Background:**

Men living with HIV in sub-Saharan Africa have sub-optimal engagement in antiretroviral therapy (ART) programs. Generic ART counselling in Malawi does not meet men’s needs.

**Methods:**

We developed a male-specific ART counselling curriculum, adapted from the Malawi Ministry of Health curriculum, based on literature review of men’s needs and motivations for treatment. We piloted the curriculum with men in six communities, with focus group discussions to assess knowledge of ART, motivators and barriers to care, and perceptions of the male-specific curriculum (n=85). We analysed data in Atlas.ti using grounded theory. We finalised the curriculum in a half-day meeting with Ministry and partner stakeholders (n=5) and implemented it in two randomized trials (IDEaL and ENGAGE). We describe the steps to develop, test and finalize the curriculum.

**Results:**

We adapted three existing topics (status disclosure, treatment as prevention, and ART side effects) and added four new topics (how treatment contributes to men’s goals, feeling healthy on treatment, navigating health systems, and self-compassion for the cyclical nature of lifelong treatment). Key motivators included: family wellbeing, having additional children, being financially stability, and earning/keeping respect. Men reported little prior understanding of how ART contributed to their personal goals, and were most interested in treatment as prevention, benefits of disclosure/social support, how to navigate health systems, and side effects with new regimens. Respondents stated that the male-specific counselling challenged the idea that men were incapable of overcoming treatment barriers to lifelong medication.

**Conclusion:**

Men need male-specific ART counselling curriculum to address their needs and increase access to and retention in HIV care. In the Malawi context, topics should include how treatment contributes to men’s goals, navigating health systems, self-compassion for lifelong treatment, and taking treatment while healthy. Other countries with high HIV burdens and limited resources could follow the steps outlined in this paper. This curriculum is being evaluated within the two randomized trials.

## Introduction

Men in sub-Saharan Africa are less likely than women to start and stay on HIV treatment, and as a result have higher morbidity, mortality and rates of viremia as compared to women. ^1– 3^ Men also have less knowledge about the benefits of starting antiretroviral therapy (ART) early and engaging in lifelong care, and how to manage treatment side effects, fear of unwanted disclosure, and other barriers to sustained treatment engagement. ^4, 5^ There are few interventions that specifically address men’s unique needs with regard to sustained ART engagement. ^6, 7^

Standard ART-related counselling in the region is not tailored to sub-population needs, including men. Unintentionally, dyadic and information-focused forms of counselling can reinforce fears about lifelong treatment through messages that focus on what clients must give up (such as alcohol, multiple partners) or do (adhere to a specific diet, and keeping to a strict schedule for taking medication) to remain on treatment. ^8^ ART-related educational materials are often biomedically focused and do not address social and behavioral benefits and support strategies for lifelong treatment. ^9^ Patient-centered, responsive counselling is needed that draws on core components of motivational interviewing, whereby counsellors collaborate with individuals to identify and strengthen their personal motivations for change. ^10^

Men face unique barriers to sustained treatment engagement that are not generally addressed in standard counselling, resulting in limited motivation to remain in care. These barriers include fear of ART side effects, perceived stigma and unwanted disclosure as well as work commitments that conflict with ART clinic hours and threaten their ability to generate income. ^11^ Many men experience general and didactic ART counselling as demoralizing and judgmental, and describe language used during counselling sessions as overly technical and paternalistic. ^12, 13^ Purely didactic counselling can also discourage clients from expressing their concerns or brainstorming personalized solutions to challenges ^14^; hence the need for motivational counselling. A growing number of studies show that male-specific ART counselling that is responsive to men’s needs and priorities can improve ART retention. ^15, 16^ Yet, there is little guidance on how to modify existing counselling curricula for targeted specific populations such as men.

We developed and implemented male-specific ART counselling and education materials for men living with HIV (MLHIV) in Malawi. The curriculum targeted men who had either never initiated ART or had initiated but had experienced treatment interruption. In this paper we describe the steps taken to develop a male-specific curriculum, modifying the existing Ministry of Health (MoH) curriculum.

## Methods

We adapted the Centers for Disease Control and Prevention’s (CDC) guidance on the adaptation process for evidence–based behavioral interventions (EBI) within the field of HIV. ^17^ The CDC guidelines propose that curriculum should be adapted in five steps, which we modified to fit the local context ( Table 1).

**Table 1.** Step by Step development of male-specific counselling curriculum.

CDC guidance on adaptation of EBI	Steps completed by our team
1. Assess needs of the population	1. Scoping review on specific needs of men for ART engagement
2. Select the intervention to be modified	2. Review MoH ART counselling materials against specific needs of men from step 1
3. Make adaptations needed based on the initial assessment	3. Adapt MoH counselling materials based on steps 1 and 2
4. Pilot the adapted intervention	4. Pilot the adapted curriculum with men 5. Review the finalized curriculum with stakeholders and implementers
5. Implement the adapted intervention	6. Implement the final adapted curriculum

Below we describe each step taken to modify the MoH curriculum into a male-specific curriculum.

### Step 1. Scoping review on men’s specific needs for ART engagement

We first conducted a scoping review of published, grey and white literature from southern and eastern Africa to identify facilitators and barriers to men’s engagement in the HIV cascade, prioritizing findings from Malawi. We conducted a search on Google Scholar and reviewed documents on men’s HIV care from stakeholders including UNAIDS, WHO and PEPFAR. ^18, 19^ The process was guided by three primary questions: what are male-specific 1) barriers, 2) facilitators, and 3) knowledge gaps affecting ART (re-) initiation and retention.

### Step 2. Review MoH ART counselling materials against men’s specific needs identified in step 1

We used findings from step 1 to identify areas in the existing MoH ART counselling curriculum where the specific needs of MLHIV identified in the scoping review were not adequately addressed. First, the lead author (MM) entered MoH counselling content by topic into an Excel data capture tool. For each topic, we entered data on the following categories: 1) What was the main message; 2) How did the content (subject, wording, structure, graphic) meet (or miss) male-specific needs; 3) What evidence supported this assessment from the scoping review; and 4) if gaps were identified, what (if any) content was supported by evidence in the scoping review (i.e. specific wording, messaging, graphics, etc.). Co-authors (KD, JH, IR) independently reviewed and gave feedback until we reached consensus on all categories.

### Step 3. Adapt MoH counselling materials based on steps 1 and 2

We adapted the MOH curriculum based on the finalized data extraction and assessment conducted in Step 2. Local Malawian team members drafted changes and/or additions to curriculum and entered these into the same Excel data capture tool. All initial adaptations were reviewed by co-authors and underwent multiple iterations. We used four in-person meetings to promote discussion and consensus building. Upon reaching consensus, the adapted curriculum was extracted from the data capture tool and formatted into a small, flip-chart style job aid for health care worker (HCW) and client use during counselling sessions, following standard formatting for counselling curriculum in Malawi. The draft male-specific counselling curriculum was translated into the local language and male-specific graphics were created by a local graphic designer.

### Step 4. Pilot adapted curriculum with men

We piloted the adapted draft male-specific counselling curriculum with 86 men of unknown HIV status between 14-18 December, 2021. We recruited men through convenience sampling from six villages in central (n=3 villages) and southern (n=3 villages) regions of Malawi. Men were eligible to participate if they 1) resided in the village and 2) were ≥15 years old. The counselling curriculum was presented in a group counselling format by two trained Research Assistants, followed immediately by Focus Group Discussions (FGD). FGDs assessed men’s post-counselling knowledge of ART, motivators and barriers to care, and perceptions of the adapted male-specific curriculum. Participants were asked if the adapted curriculum was relevant to their lives and their motivations as men. They were asked to identify any topics in the curriculum that were unclear or did not motivate them, and to raise any additional needs or questions they still had about HIV services. They were also invited to suggest any further modifications including specific language and graphic choices to fit the local context. FGDs were conducted in the local language (Chichewa), translated to English, transcribed, and analyzed in Atlas.ti8 ^20^ using grounded theory. ^21^ Participant responses were reviewed, main themes summarized, and the curriculum further modified to incorporate feedback. Verbal informed consent was obtained prior to the interviews. We administered verbal consent to minimize risks generated by written consent. This was approved by the ethics board prior to data collection.

### Step 5. Review of finalized curriculum

The male-specific ART counselling curriculum was reviewed by two groups: programmatic stakeholders (implementers and researchers) in Malawi and HCWs (nurses and lay cadre treatment supporters) who would implement the new curriculum.

*Stakeholders:* In a one-day stakeholder workshop, the updated male-specific counselling curriculum flip-chart and artwork were presented to PEPFAR implementing partner staff (n=5) and HIV researchers (n=6). Participants reviewed the content (including topics, analogies, wording, and graphics) to ensure the curriculum complied with MOH guidelines for HIV counselling and assess the curriculum’s feasibility and acceptability among stakeholders. The curriculum was then modified based on consensus.

*Implementers:* Finally, male nurses (n=10) and a lay cadre (treatment supporters, n=10) were trained in the new curriculum and invited to provide further modifications to improve clarity and implementation. The training took place over a five-day period and all modifications were agreed upon by consensus. Final edits were made prior to implementing the male-specific counselling within the parent trials.

### Step 6. Implement the final adapted male-specific counselling curriculum

The final male-specific curriculum was implemented as part of two randomized control trials (IDEAL and ENGAGE). Results of the implementation will be described in in the trials’ primary outcome papers.

## Results

### Step 1: Scoping review on specific needs of men for ART engagement

We identified eight overarching male-specific barriers and facilitators affecting men’s ART re-initiation and retention ( Table 2). First, men wanted to be treated as equals with HCWs, valuing respect and autonomy over care. ^11, 22^ Men responded poorly to shouting or conflictual interactions with HCWs, or counselling sessions in which they were simply given instructions without being given motivational explanations or choice. ^23– 25^ Men wanted to have interactive counselling sessions, and to be treated as equal participants in their own health care. ^25^ They needed to have a sense of ownership and agency over their own health. ^16^ Without positive interactions with HCWs, men often opted to avoid services altogether in order to maintain respect and autonomy. ^22^

**Table 2.** Barriers and facilitators affecting men’s ART (re-)initiation and retention on ART in Malawi.

No	Theme	Barrier/Facilitator	Description
1	Autonomy	Facilitator	Men want to be engaged in and have ownership over their HIV care
2	Masculinity	Barrier	Expectations, norms and roles influence men’s decisions about starting treatment, disclosing their status and remaining in care
3	Men as economic Pillars/competing interests	Barrier	Men see themselves as economic pillars, they want to see the value of ART in their income-generating activities
4	Good health	Facilitator	Men want to have good health to maintain their strong physical appearance and reputations in the community
5	Gendered service provision/preferred gender matching service provider	Barrier/Facilitator	Men are less familiar with health systems/Men would use the services if provided by HCWs of their own gender
6	Good health Limited ART knowledge/Side effects	Barrier	Knowledge gaps on TASP, side effects and flexibility about taking ART
7	Social support/guardian support	Facilitator	Men perform better on treatment if they have a role model, and support, especially from a fellow man
8	Self-compassion	Facilitator	Clients who have self-compassion are likely to have better adjustment to treatment, less stress, positive health seeking attitudes, and are more likely to disclose status and practice safe sex

Second, extensive literature framed men as the economic pillar of the household. ^26, 27^ Bearing the responsibility for income generation was both a barrier and facilitator for men, particularly in the context of poverty. Income generation acted as a barrier because men had to interrupt treatment due to travel for work ^5, 28, 29^ and conflicts between clinic and work schedules. ^11, 30^ Failing to provide for their families was widely interpreted as a weakness, directly undermining men’s internalized (and socialized) expectations of masculinity. ^27^ However, income generation could also act as a facilitator to men using ART services, as men could clearly see how ART contributed to their earning potential and ability to provide for their families. ^31– 33^

Third, men were motivated by maintaining their own health. ^31, 34^ Health was important from several perspectives: good health allowed men to keep their HIV status private from community members, maintaining their reputation in the community. Good health was also associated with a strong physical appearance which increased men’s options for sexual partners. ^27, 35^ Finally, physical strength and vitality were seen as directly influencing men’s earning potential, key to their current and future role as provider for their family. ^2, 7, 35, 36^

Fourth, the gendered organization of HIV services means that men are less familiar with health facility protocols and strategies to overcome facility-level barriers to care. ^25^ Men often reported difficulties navigating health facilities, especially as the majority of services provided at facilities is designed for women and children. ^7, 16, 37, 38^ Many men preferred to be offered HIV services by male HCWs, and studies have found that gender-sensitive services can facilitate men’s service utilization. ^27^

Fifth, men had limited knowledge about ART services. ^5, 36, 39^ Men were still largely unaware of Treatment as Prevention (TasP) and the benefits of early ART initiation, ^5^ and had limited understanding about drug doses and how HIV and ART work within the body. Men over-estimated the risk of severe side effects when taking ART, leading to concerns that ART would undermine their health. Men were also concerned that such side effects might impact their physique, a key barrier for men’s slow uptake of ART services. ^40, 41^ While side effects are rarely experienced with new dolutegravir-based regimens, ^42, 43^ the legacy of side effects due to old regimens (such as nausea, bad dreams, and fat deposits) still informs men’s fears of treatment today. ^44^

Sixth, social support from family members or male friends was critical to men’s sustained retention in ART services. Fear of disclosure of one’s status and the subsequent anticipated stigma is a common reason for defaulting from ART. ^11^ Recent studies suggest that having a male role model who has successfully engaged in ART and is thriving in care may be particularly motivating for men. ^25, 30, 45^

Seventh, studies found that self-compassion with life-long treatment resulted in better adjustment and lower stress, anxiety and shame. ^32, 46^ Self-compassion facilitates HIV status disclosure, encouraging clients to practice safe sex and seek medical care. ^46^ We defined self-compassion as being able to acknowledge the struggles one faces and celebrate the small gains achieved along the journey. However, men may have limited self-compassion as harmful gender norms define men as being strong and self-sufficient, and never being weak. ^27, 32^ Self-compassion with lifetime treatment may help alleviate fears that prevent some men from ever starting treatment and may remove the feeling of being overwhelmed by treatment.

Finally, the literature highlights the huge role of masculinities in men’s engagement in treatment, which needs further exploration. ^5, 27, 47^ A meta-analysis found that beliefs about masculinity may influence treatment outcomes. ^48^ For example, in South Africa, men considered being tough, unemotional, aggressive, denying weakness, sexually unstoppable and appearing physically strong as important male traits. ^27, 32^ These masculinity constructs are likely to prevent some men seeking health care services. ^26, 32, 39^

### Step 2: Review MoH counselling materials for gaps in reaching men

We found that the MoH ART counselling curriculum was didactic and directive. The curriculum was largely biomedical-focused, explaining what ART does and the need for adherence, but not explicitly linking how ART can contribute to individuals’ goals and needs (see Tables 3a and 3b for the completed data capture tool). The curriculum did not provide explicit probes needed to facilitate interactive or client-centered counselling. The MoH curriculum did not address many of the specific barriers and facilitators experienced by men.

**Table 3a.** Modified topics in the adapted counselling pamphlet.

MOH generic counselling messaging	Men specific gaps	Suggested changes	Specific change: Messaging	Specific change: Images
• Overall: focused on biomedical HIV/AIDS and management	• Does not address the non-biomedical barriers • Not interactive	• Link all HIV/AIDS and ART messaging to men’s goals and priorities • Ask questions and solicit men’s input	• Use motivational counselling approaches: counsellor guides client to identify practical solutions	• (Re) develop imagery that is context specific and relatable to adults
• Introduction, requirements of a guardian presence, confirmatory testing of counsellor, introduction of ART and HIV services	• MOH sessions are classroom-like, where counsellor shares information to client; not interactive	• Demonstrate that men are not alone, include HIV/AIDS prevalence rates • No guardian requirement; importance of guardian discussed as stand-alone topic	• Adopt motivational interviewing techniques	• (Re) develop imagery that is context specific and relatable to adults
• Health and ART: Describes immune system biomedically and how ART protect the immune system	• Biomedical approaches: Messaging and imagery does not link immune system, HIV & AIDS and opportunistic infections to direct gains in business, family and future	• Link HIV/AIDS messaging and imagery, body immunity and opportunistic infections to the goals and ambitions mentioned by clients • Experience-based counselling	• Good health due to ART leads to social-economic gains	• A man with a shield fully covered by arrows (symbol of opportunistic infections)
• Who can get HIV, how strong is immunity in adults vs. children, how can one tell who is HIV-positive; staying healthy while positive	• Messaging is to a general population; does not target special population	• Include HIV prevalence rates in adults; messaging to focus on men	• ART enable men to achieve goals like care for family, business, agriculture and looking good to maintain community respect	• (Re) develop imagery that is context specific and relatable to adults
• How ART work: the type of medicine and how it works, how to take ART, benefits of ART; viral load (VL) and ART	• Focus is on biomedical benefits • Compounded topics: VL and treatment as prevention, VL testing and adherence. Did not address men’s barriers like pill burden, treatment of opportunistic infections, poor adherence, what to do when one forgets to take a daily dose of ART	• Focus impact of ART on body outlook, social and economic benefits, and other motivators • Add as separate topics: VL, treatment as prevention, pill burden, what to do when one forgets to take ART, adherence, address barriers and develop action plans	• ART cannot saturate in the body, make the virus in men weaker and makes body stronger enabling them to work and provide • ART do not work alone: CPT, 3HP and IPT help to fight opportunistic infections (drugs are explained in detail, dosage and importance)	• Add actual images of ART, CPT, 3HP and IPT • Add new images explaining low and high VL
• Prevention of transmission from mother to child and how to prevent the transmission of HIV so that you remain healthy	• MOH stresses role of women; role of men not explained	• Identify men’s role and explain to men how prevention works • Add a new topic on the roles of men	• Men have huge role in preventing transmission to babies (taking ART correctly and achieving low VL)	• Father holding baby in his hands and father leading his son to brighter future
• Preventing transmission of HIV to remain healthy	• Does not address men’s knowledge gaps; condom use vs. treatment as prevention and ability to bear children while HIV positive • Does not discuss treatment as prevention and early initiation	• Link condom usage to taking of ART, treatment as prevention and discuss proper condom use	• ART lessen the risk of infection; the only way is to use condoms. Try to use condoms and reduce number of sexual partners. Discuss with them how condoms support ART to prevent transmission and re-infection. Ask client to explain how they can wear a condom and correct them	• New image linking a man, ART as a shield and the man’s partner
• Mild side effects of ART within the first month and serious side effects	• Old regimen side effects discussed; side effects not discussed in relation to the benefits of ART hence need for men to see beyond side effects	• Discuss old side effects vs. new regimen side effects • Emphasize that most side effects are not severe and there is medical help readily available • Highlight benefits of ART and the need to overcome fear of side effects	• In 2019 new ART were introduced with VERY FEW side effects that last between 2-3 days • Your provider will help you if you have side effects • New regimen has fewer side effects and is crucial to achieving men’s goals	• Show old vs new ART regimen

**Table 3b.** Newly introduced topics in the adapted counselling pamphlet.

Missing in MOH generic counselling messaging	Men specific gaps	Suggested changes	Specific change: Messaging	Specific change: Images
• Treatment as prevention	• MOH focus is on condom use and reducing number pf partners	• Explain how treatment as prevention works	• If you take ART daily, it is unlikely that HIV will be passed to your partner	• (Re) develop imagery that is context specific and relatable to adults
• If you forget taking your ART	• MOH is rigid and stresses taking ART same day and same time	• Explain to the client the scientifically allowed time to take ART	• If you miss taking ART, take immediately; if more than 12 hours late, do not take ART, take the next day	• Picture of man worried about having missed taking ART, next to a man taking ART
• Experiences with health facilities	• MOH does not address facility level barriers	• Introduce facility client services like health advisory committee; highlight client’s rights and responsibilities	• You seek redress form Health Advisory Committee, a different provider or seek transfer out	• Men on waiting area with HAC on the wall
• Male guardian support	• MOH stresses on general guardians the importance of having a male guardian; role of male guardian not highlighted	• Discuss role of male guardian and identify who can be the client’s male guardian	• With client, identify which male guardian would be central to their treatment	• Different scenarios of a man helping another man
• Alcohol	• MOH stresses on stopping alcohol; does not explain why clients must stop and what to do when struggling with alcohol	• Assess client’s knowledge and habits; discuss how alcohol might impact efficiency of ART	• Even if you don’t plan to stop taking alcohol, start taking ART	• Scales of balance of taking one bottle of alcohol vs consuming more
• Mobility	• MOH barely discusses mobility and ART, emergency refills and adherence	• Discuss emergency refills and the requirements for travelling	• Know your regimen, you can get emergency refills at any facility, you can get a refill before your appointment date	• A man packing for a trip, with ART and health passport book on the items to be packed
• Self-compassion and treatment	• MOH does not address challenges in the lifelong/cyclic treatment journey	• Acknowledge decision is difficult and journey has challenges • Establish that there is a way to manage barriers and there is support to slowly but steadily achieve	• Taking ART for life is challenging, but slowly one can learn and overcome challenges	• A picture of a provider helping a client climb a mountain

There was little consideration of ART in relation to men’s overarching goals and fears. Another gap in the curriculum was the limited discussion on social support. Lastly, the curriculum presented a strict approach to ART adherence, without explaining that missed doses could be taken subsequently and did not include any information about self-compassion while navigating lifelong medication, or any Welcome Back messaging for individuals who may fall out of care in future. Based on this assessment, multiple messaging and graphic changes were suggested.

### Step 3: Adapt MoH counselling materials

The draft counselling curriculum adapted to male-specific needs included four new themes and three modified themes ( Tables 3a, 3b & 4). Key messaging included ART as a way for men to regain health, provide for their families, maintain their role within their community, and have self-compassion with their own ART adherence and facility attendance as life is complicated and lifelong adherence can be difficult.

**Table 4.** Modified and new themes in the male-specific counselling pamphlet.

Theme	Male-specific modification
New themes	
How treatment contributes to men’s goals	We work with men to frame their possible future goals based on their health. Prior to this, men argued that ART engagement competed with business and agriculture priorities. We discuss how HIV services contribute to their key goals. We employ motivational interviewing skills to discussing ART as an integral component to achieve goals.
Feeling healthy on treatment and low ART knowledge	We explicitly acknowledge challenges of taking ART when feeling healthy, and ask men to reflect on their own experience. We discuss how taking ART while healthy will prevent disruption of earning prospects and support strong business and better families, using local analogies and graphics that resonate with Malawian men.
Navigating health system	We discuss barriers men have experienced when seeking health services at facilities and discuss how to overcome them, and notably, to report poor services. We address the pre-existing discord between HCWs and men.
Self-compassion/patience for lifelong treatment	We normalize the fact that men may forget a dose, feel guilty and panic. We highlight the importance of returning to care as soon as possible. We discuss alcohol use and fears about long-term treatment adherence. We acknowledge individual concerns about competing responsibilities, fear of disclosure, and treatment fatigue, and stress that such fears are normal.
Modified Themes	
Status disclosure to male friends/family	Beyond disclosure to their sexual partners, we highlight the importance of disclosing to their male social support system. We discuss who is important to them, how to disclose to other men, and how to get social support for ART disclosure.
Treatment as prevention	In addition to the general treatment as prevention message, we provide a detailed description about how treatment as prevention works, men’s role in preventing vertical transmission, and how these concepts contribute to men’s role as provider and building a stronger future.
Understanding ART (Side effects)	MOH counselling simply describes potential side effects and tells clients to report to clinic if they persist. We focus on the changes in ART regimen including that the new dolutegravir regimen has fewer side effects than the older regimens.

The counselling curriculum was printed on a small, flip-chart job aid. In developing this, the artist featured a central male character. The character was intended to be relatable as a Malawian man and a role model figure. He was strong and clean but simply dressed like a wealthy man living in the village, and middle-aged (indicated by a few grey hairs). The male character was depicted in various scenarios that were identified as motivating in the scoping review. For example, being healthy and providing for one’s family were depicted through the image of the main character farming and looking strong.

### Step 4: Pilot modified male-specific counselling curriculum

In piloting the male-specific counselling curriculum, men believed that the new curriculum directly connected with what they cared about: their families’ well-being and their role as provider.

*“It [the curriculum] is connecting to our income generating activities because [it makes it clear] that if we are HIV positive and we adhere to the treatment, we can be strong enough to do our various works and make money in the process and take care of our families.”- FGD, 30-40 Years, Married, Businessman*.

Men appreciated the fact that the new counselling was interactive and solicited men’s voices, and contrasted this experience with the directive and authoritarian counselling styles to which they weare accustomed. Men did not like the classroom approach of standard counselling and wished to be fully informed and involved in interactive discussions and decision-making about their health.

Men reported numerous curriculum topics that they found motivating. Most men were unaware of TaSP and were highly motivated by the fact that taking ART meant that men living with HIV could have HIV-negative children.

*“A new thing that I have learnt today is that if we are expecting a child and we both are HIV positive, taking the treatment as recommended makes sure that the baby to be born would not get infected. I will even teach others about it.”- FGD, 20-30 Years, Married, Barber.*

Men were also motivated by hearing that the new ART regimen (dolutegravir) had reduced side effects. Prior to the counselling, many men still associated ART with serious side effects that could inhibit work and income generation, especially if taken while a man was still healthy. Most men believed that the advances of dolutegravir, including reduced side effects and drug toxicity, needed to be thoroughly communicated to men in the community to improve men’s acceptance of treatment.

*“We are happy with the messaging on ART [in the new curriculum] because some of us were concerned that when you start taking the ART you have a lot of side effects. But today we learnt that the new drugs have no side effects and one can take them without any problems.”-FGD, 30-40 Years, Married, Businessman.*

Men were also largely unaware that there was any flexibility about the time when they took ART. Most men believed that there were very strict timelines for taking daily doses. They were unaware that if they missed a dose, they could still take their dose within 12 hours. This flexibility was important because it helped men feel like they could reach “good enough” adherence without having to restructure their lives around taking HIV treatment, making adherence attainable for busy men with competing priorities.

*“The new thing that I have heard is that in the past we had knowledge that people on medication take them only at night, but we have learnt today that they can be taken at any time of the day.”- FGD, 40-50 Years, Married, Businessman*.

**Figure 1.**
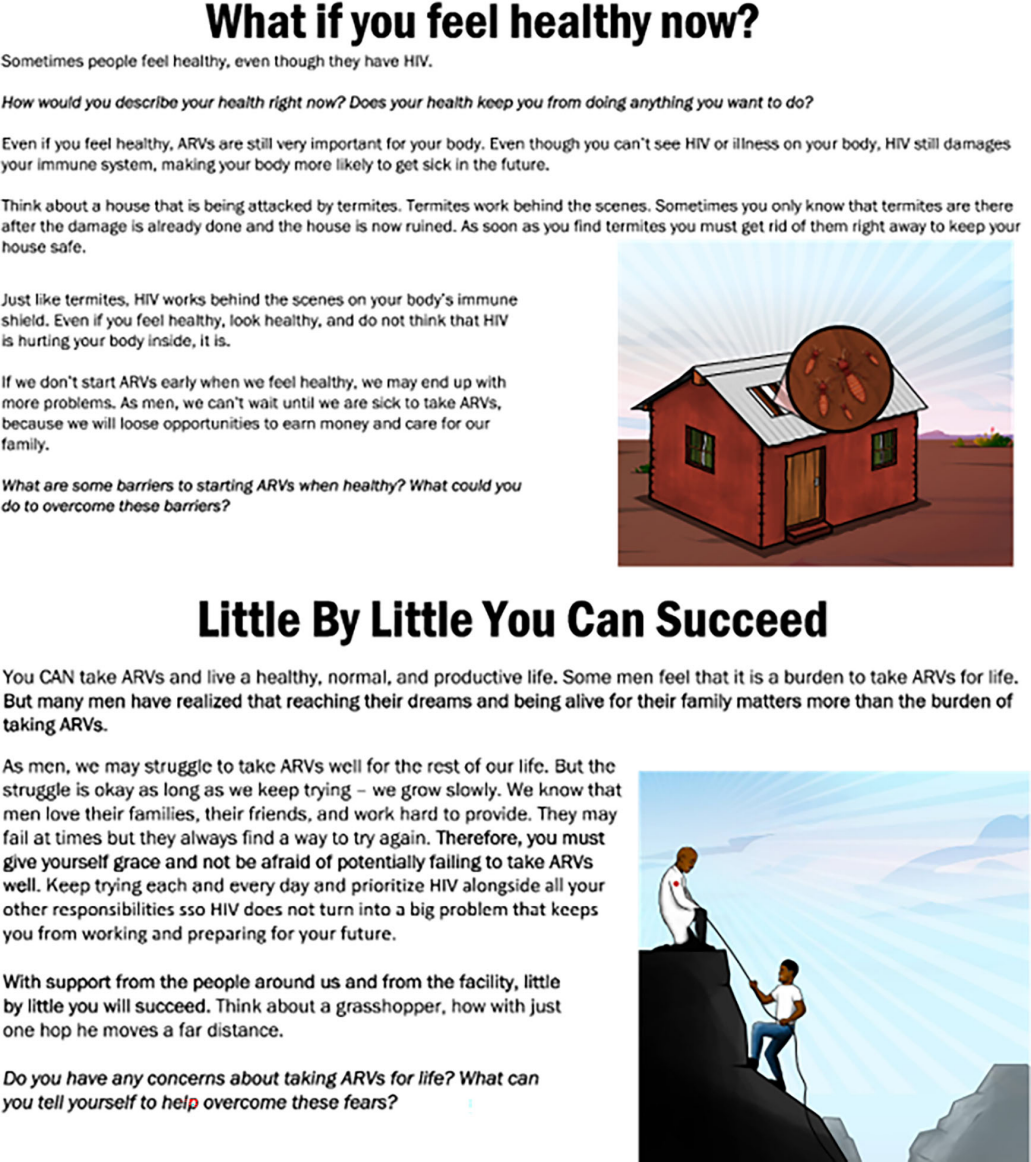
Slides on Taking ART while healthy and Self compassion topics.

Men had several suggestions to further refine the curriculum. First, they suggested that local analogies and popular phrases be used throughout the curriculum so that men could easily relate to the topics. For example, in the topic “taking ART while healthy” men suggested using the analogy of a house that is slowly being attacked by termites (the virus in a healthy body), which, if not treated in time, will fall completely (the development of the stage of AIDS). For the topic, “Lifelong treatment”, they proposed an analogy of the persistence of a grasshopper which slowly hops (small gains day by day) to cover long distances (see Figure 1).

**Figure 2.**
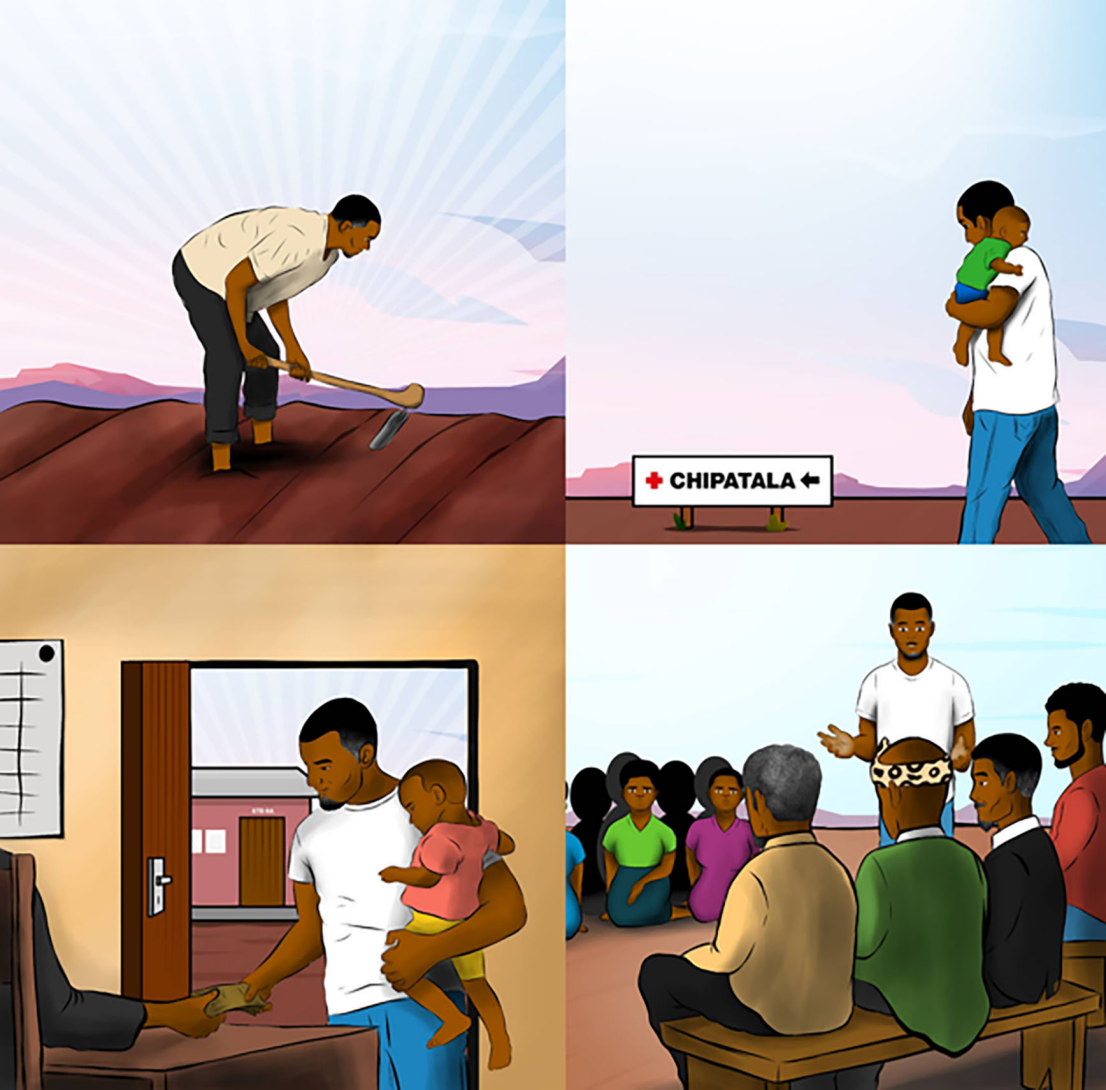
An image showing men as pillars of the society and homes.

Men also desired graphics throughout the counselling job aid that included men they could relate to ( Figure 2).

*“I think the pictures would also help because at school when we are teaching a child we use pictures to illustrate what is going on and how to identify things. Pictures would help people to understand the benefits.”- FG, 20-30 Years, Married, businessman.*

*“Give us a picture showing the drugs so that we should be able to show some men can they can see the difference between the old and new medication.”- FGD, 30-40, Married, farmer.*

Based on the FGD’s, the curriculum was further adapted, with an increased emphasis on TasP, reduced side effects of the new ART regimen, ART ability to support men’s overarching goals, and the inclusion of local analogies and graphic visualizations of relatable men.

### Step 5: Stakeholder review of finalized curriculum

Stakeholders and implementers made further small modifications to content and graphics. Both stakeholders and implementers were concerned about the emphasis on TaSP, fearing that it may increase risky sexual behavior. After multiple rounds of discussion, we minimized TaSP messaging and removed language that did not explicitly encourage condom use. A near-final version was reviewed with facility and program staff, nurses and lay-cadre.

### Step 6: Final product and implementation strategies

**Figure 3.**
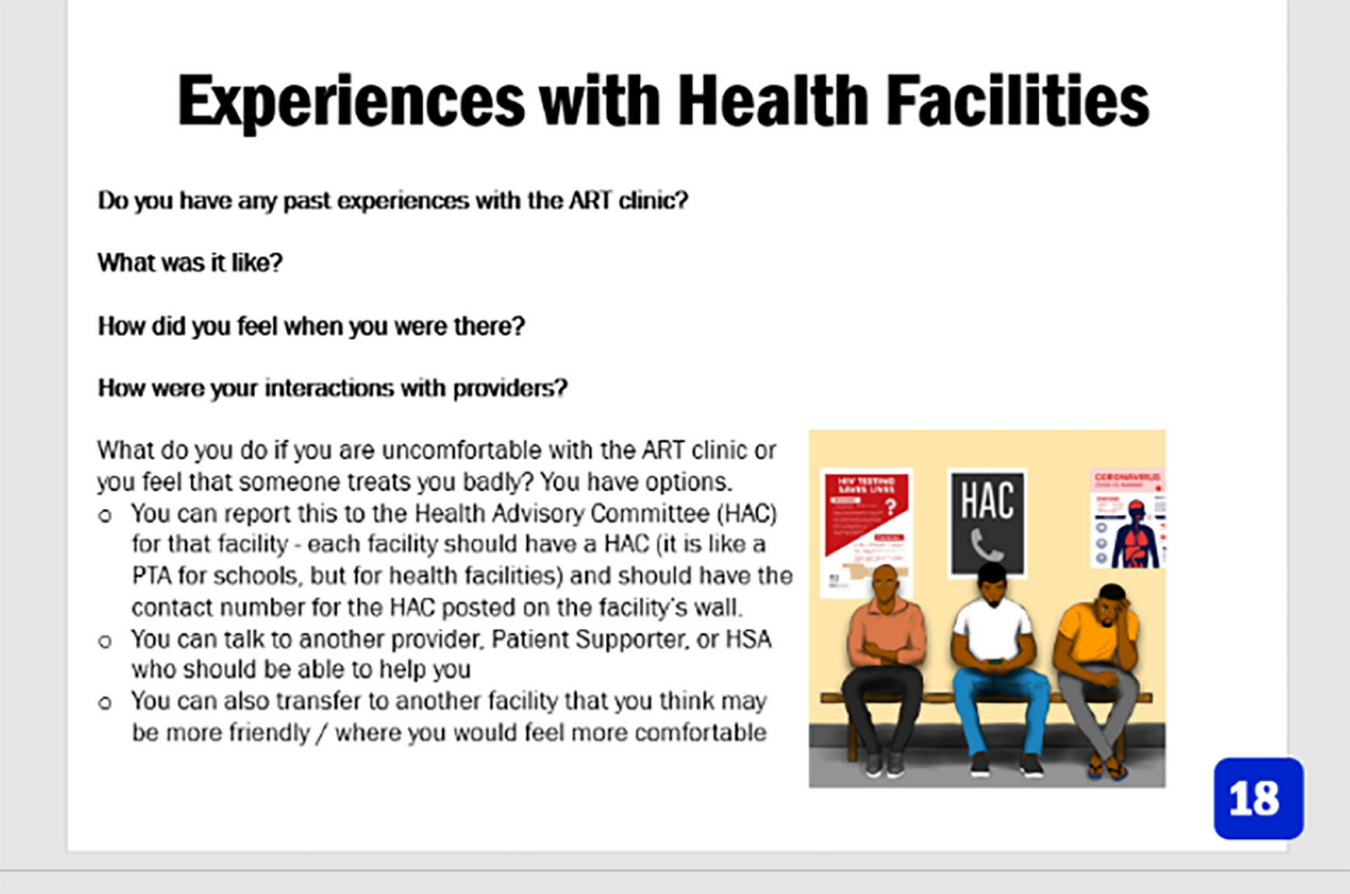
An example for the final product page that contained probes and open ended questions.

The final product was a 17-topic counselling pamphlet. The counselling materials included a script for every topic with key bullets to guide HCWs, and probes that promoted client-centered approaches to counselling, including open-ended questions and motivational explanations for how the topic may help men achieve their life goals ( Figure 3).

## Discussion

ART counselling curricula must be tailored to address specific barriers and build on facilitators relevant to sub-populations. We used a five-step process to develop ART counselling specifically for men in Malawi, using men’s own words and addressing men’s unique needs. We identified the needs of men, gaps within standard counselling curriculum, and how to adapt standard curriculum. We aimed to meet men’s needs in ways that were accessible and motivating to men, being responsive to their local context. We found that adapting standard ART counselling to men’s specific needs in Malawi was feasible and highly acceptable to men. We found that men needed to be treated as equals and engaged in planning and deciding on their health. However, they had limited knowledge on topics like how treatment contributes to their goals, navigating the health system, and self-compassion while on treatment. Standard counselling curriculum did not include these topics or person-centered counselling techniques essential to building trust between HCW and client ^49– 51^ and self-efficacy within clients. ^52, 53^ Importantly, we found that men in Malawi had large gaps in knowledge regarding TaSP, the benefits of dolutegravir and early ART initiation. Despite this, they were keen to know more about how to live well with HIV. Men suggested that the ART curriculum should incorporate local analogies and graphics representing local men in Malawi.

Counselling must promote and value individual autonomy and agency. In our study, men welcomed and accepted interactive, equal relationships with HCWs when they sought health services. In a previous study in Malawi, ^25^ men expressed the need for counselling curricula that recognized them and allowed for their expression of thought and participation in health-related decisions. Men may also experience poor outcomes due to HCW bias against men as difficult clients. ^54, 55^ Some HCWs believe that men do not need extra help engaging in HIV services but actively choose not to access care as they are too stubborn and powerful. ^55, 56^ Such perceptions can reduce HCW motivation to actively engage men in counselling. A less didactic design, flexibility, and allowing clients to ask questions and debate information, would support the development of tailor-made sessions that respect men’s autonomy and allow them agency in decision making. This could improve men’s uptake of ART services and their adherence on ART.

Many men had major gaps in their knowledge about ART and the health system. ^5, 7^ Men were largely unaware about TaSP, its benefits and how it can contribute to their goals of a healthy family. TasP seemed a fairly new approach to men and current MoH counselling curriculum has not yet been updated to include the concept. This curriculum, with its strongly biomedical approach, does not include TasP or the benefits of early treatment in a manner that appeals to men’s needs. Including TasP messaging in the counselling curriculum not only addresses the knowledge gap, it acts as a motivator, resonating with men’s priorities to provide and protect their family. ^7, 36^ Facility navigation was another barrier to men’s engagement with health facilities. ^25^ The MoH curriculum did not include discussions on how men may deal with health system barriers including unsatisfactory care from providers. Including messaging about navigating health facilities in counselling curricula would familiarize men with the health system, thereby facilitating early treatment and adherence and addressing a health system barrier to care. ^7, 57^

Self-compassion should be central in the male-centred counselling curriculum. Previous studies have identified lack of emotional readiness as a key barrier to men’s initiation and retention in ART care. ^11^ This may compound other challenges like stigma and fear of losing relationships, which also affect adherence. ^58^ In the context of ART, it has been argued that emotional readiness - an individual’s identification that treatment is beneficial - is an important predictor of adherence and retention. ^59^ The current MoH curriculum does not explore individual readiness, nor does it promote self-compassion to address individual-level barriers. A curriculum for male counselling must therefore promote individuals’ emotional readiness, status acceptance and self-compassion in their life-long treatment journey.

In order to be client-centered, the counselling curriculum should include pictures, prompts and job aids. ^60– 62^ A recent review of medication argued for the use of images instead of didactic methods that depend on verbal and oral communication with patients. ^63^ The absence of client-centered counselling approaches affects everyone. It reduces the impact of counselling for both male and female clients. ^64^ Additionally, it can undermine HCW job satisfaction as there are few positive, meaningful interactions and reciprocity with their clients. ^65, 66^ With a biomedical and didactic approach, the current MoH counselling fails to provide client-centered counselling. It offers generic educational information and has less flexibility to adapt care interventions that utilize men’s motivators, meet their needs and address their barriers.

Our study demonstrated the feasibility of modifying a national counselling curriculum for the needs of a specific population. A limitation is that we focused broadly on men, but did not consider sub-populations of men, for example by age, sexual orientation or levels of absolute poverty. These factors may impact on health-seeking behaviors and attitudes towards HIV services, ^26, 35, 41^ and certain populations may require further tailored counselling curriculum. Incorporating client-centered counselling ^67, 68^ and motivational interviewing techniques can help address this limitation. However, HCWs still need to be trained on unique strategies to successfully engage sub-populations of men.

Finally, we believe that the approaches discussed in this paper are applicable to other sub-populations, other HIV approaches (e.g. HIV prevention) but also with other non-communicable diseases.

## Conclusion

In conclusion, we found that it was possible to adapt a curriculum to suit men’s needs, thus addressing barriers to ART and support treatment initiation and adherence. Adapting a curriculum requires in-depth understanding of target users and piloting the curriculum with target population.

## Compliance with ethical standards

The study protocol was approved by the institutional review boards of the University of California, Los Angeles (UCLA) (#21–000592) and the National Health Sciences Research Council (NHSRC) (#2562) in Malawi. The trial is registered with ClinicalTrials.gov as NCT04858243. There are no potential conflicts of interest to declare.

## Disclaimer

The content is solely the responsibility of the authors and does not necessarily represent the official views of the National Institutes of Health or the Bill and Melinda Gates Foundation.

## Data Availability

We are unable to share these data in a public repository because there may be identifying or sensitive participant information. Although the data are de-identified, the information about where the study was conducted may draw attention to individual ART clients. Additionally, a limited number of health care workers specialize in HIV care within participating facilities. Individuals familiar with these facilities may be able to identify specific health care workers by characteristics such as age, sex, number of years working at the facility, or by particularly memorable stories from the facility. Malawi’s National Health Sciences Research Committee approved our protocol in which (under the ethical considerations section on protection of human subjects’ privacy and confidentiality) we stated that participant information will not be released without written permission from the researchers except as necessary for review, monitoring, and/or auditing. These restrictions have also been imposed by the UCLA institutional review board. We are thus able to share code summaries, but not transcripts in the original form. For any data requests, please contact the lead author, Misheck Mphande ( misheck@pihmalawi.com), who will liaise with the PIH/UCLA study team for permission to release deidentified data.
